# Mutual Coupling Reduction of a Multiple-Input Multiple-Output Antenna Using an Absorber Wall and a Combline Filter for V2X Communication

**DOI:** 10.3390/s23146355

**Published:** 2023-07-13

**Authors:** Yuanxu Fu, Tao Shen, Jiangling Dou

**Affiliations:** Faculty of Information Engineering and Automation, Kunming University of Science and Technology, Kunming 650032, China; shentao@kust.edu.cn (T.S.); Jianglingdou@kust.edu.cn (J.D.)

**Keywords:** MIMO, V2X, combline filter, absorber wall

## Abstract

This paper presents an MIMO antenna for vehicle-to-everything (V2X) communication, which adopts two ways of combline filters and absorption wall decoupling. A combline filter and an absorption wall are used, respectively, for internal and external decoupling. The combline filter is incorporated between the ground of the two adjacent antennas, which reduces the mutual coupling between them. Additionally, the mutual coupling of radiation between adjacent antennas is significantly reduced by the absorber wall. These combline filters and absorber walls use the method of electromagnetic field distribution to explain the reduction in the mutual coupling between the adjacent antennas. The transmission coefficient and surface current distribution explain the effectiveness of the decoupling structure. When the frequency is between 3.8 and 4.8 GHz, the simulation and measurement results show that $S_{11}$ is less than −10 dB, the bandwidth is 25% and the peak gain is 7.8 dBi. In addition, the proposed MIMO antenna has a high isolation between antenna units (>37 dB), and the envelop correlation coefficient (ECC) is less than 0.005.

## 1. Introduction

The fifth generation of mobile communications (5G) will meet the growing demand for high-speed data transmission, large capacity and ubiquitous connectivity, as the global positioning system (GPS) [1,2], satellite digital audio radio service (SDARS) [3,4], cellular communication [5,6,7] and vehicle-to-everything (V2X) communication [8,9] meet the needs of navigation, communication and entertainment. As the amount of information sources increases, V2X communication will become more crowded and there will be a faster fading issue, which leads to a decline in efficiency. As a result, multiple-input multiple-output (MIMO) antennas have been proposed to improve the channel-capacity-limited spectrum resources [10,11,12,13]. However, the coupling interference of MIMO antenna systems causes the radiation performance of a single antenna to decrease. Recently, several low coupling design techniques have been proposed to reduce the mutual coupling of adjacent antennas. The details are as follows: defective ground structure (DGS) [14], parasitic element [15], electromagnetic band gap (EBG) [16], neutralization line [17] and decoupling network [18] methods. Some of these methods require the antenna spatial distance, and others degrade the radiation of MIMO antennas or require complex construction MIMO antenna designs. To solve the these problems, the coupling of antenna elements is reduced by metamaterials. In [19], a Taichi-Bagua-like metamaterial with a single layer substrate has achieved high isolation. In [20], ENG metamaterial structures have achieved isolation enhancement. In [21], the isolation of MIMO antennas was improved by a complementary split ring resonator CSRR metamaterial.

To improve the isolation of MIMO antennas, an absorbing metamaterial is proposed as a new platform to reduce the coupling of antenna elements. Currently, there are two ways to isolate MIMO unit antennas. One method uses planar isolation to reduce the coupling of surface currents and thus reduce the coupling between antennas [22,23]. The other method uses a three-dimensional absorption wall, which mainly isolates the radiation coupling of the antenna [24,25]. However, such absorbent materials do not meet the requirements of miniaturization, and few multiple technologies methods are used to reduce the coupling of MIMO antennas.

In this paper, the absorption unit size of the absorber wall is only 13 × 13 ${\mathrm{mm}}^{2}$, and the coupling of the MIMO antenna is reduced by a combline filter and absorption wall. These combline filters and absorber walls use the method of electromagnetic field distribution to explain the reduction in the mutual coupling between adjacent antennas. The transmission coefficient and surface current distribution explain the effectiveness of the decoupling structure. The rest of this paper is as follows. Section 2 illustrates the MIMO antenna configuration and design method. Additionally, the design procedure of the proposed combline filter reduction techniques is explained in detail in Section 3. Section 4 presents a concrete analysis of the absorption wall decoupling principle. Section 5 mainly describes the S parameter, MIMO isolation, antenna gain, antenna radiation efficiency and envelope correlation coefficient (ECC) measurement and simulation results. The conclusions are provided in Section 6.

## 2. Configuration

**Figure 1:**
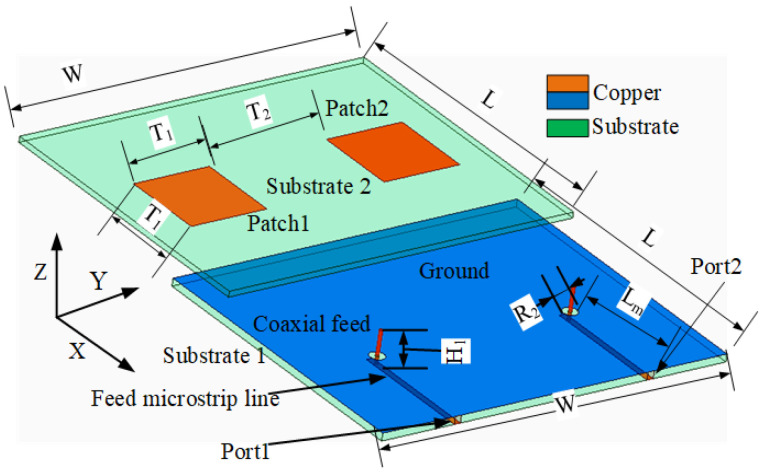
Geometry of the MIMO antenna.

**Figure 2:**
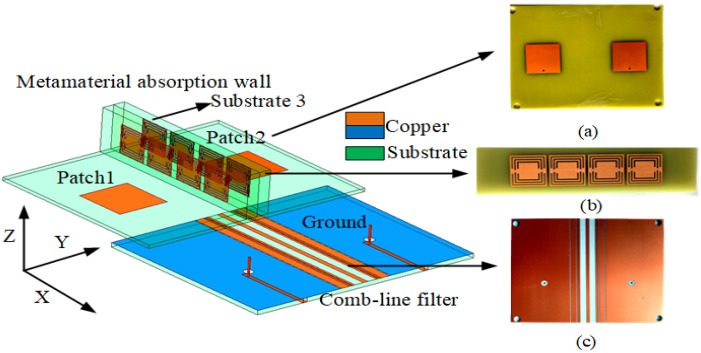
Design and fabrication of an MIMO antenna with a combline filter and absorber wall. (**a**) Radiation layer fabrication. (**b**) Absorber wall fabrication. (**c**) Ground plane and feed port fabrication.

Figure 1 represents the geometry of the MIMO antenna. The antenna structure comes from reference [26], which has a low profile, high efficiency and high gain. It consists of a two-layer substrate connected by a coaxial feed. Substrate 1 and 2 adopt the Ro4003 material ($\epsilon_{r}$ = 3.55 and tan δ = 0.0027) with a thickness of h = 1.524 mm and the FR-4 material ($\epsilon_{r}$ = 4.4 and tan δ = 0.02) with a thickness of h = 1.5 mm, respectively. A 50 ohm coaxial cable is used to feed. The antenna has a favorable radiation efficiency and gain. The isolation can reach 20 dB. The antenna parameters are as follows: W = 100 mm, L = 80 mm, $T_{1}$ = 22 mm, $H_{1}$ = 5 mm and $L_{m}$ = 33.5 mm. The diameter of the isolation hole between the feed and the ground is $R_{2}$ = 4 mm. Figure 2 shows the mutual coupling reduction of the MIMO antenna using the absorber wall and the combline filter, which is based on adding combline filtering and a metamaterial absorption wall to the structure in Figure 1. The combline filter belongs to the internal decoupling structure and the absorber wall is the external decoupling structure. The combline filter has a simple structure and cannot affect the radiation and efficiency of the antenna. Figure 2a–c shows the physical components of the MIMO antenna with a combline filter and absorber wall as follows: (a) is the radiation layer; (b) is the combline filter; and (c) is the ground plane and feed port. The combline filter is composed of seven microstrip lines, which are added between adjacent antennas to achieve adjacent antenna decoupling. The absorption wall substrate 3 adopts an FR-4 substrate with a thickness of 5 mm, which includes four absorption units arranged on both sides to absorb radiation to improve the isolation of the adjacent antenna. The following is the specific analysis process of decoupling between the two methods.

## 3. Ground Adopts a Combline Filter to Reduce Antenna Element Coupling

We analyze the effect of the combline filter to reduce the coupling of the MIMO antenna from three aspects. First, the decoupling and bandwidth of the original MIMO unit are compared with those after combline filtering. Second, a theoretical model and equivalent circuit are used to calculate the specific parameter values of the MIMO antenna of the cofilter. Finally, an electromagnetic simulation verifies the decoupling capability of the combline filter.

**Figure 3:**
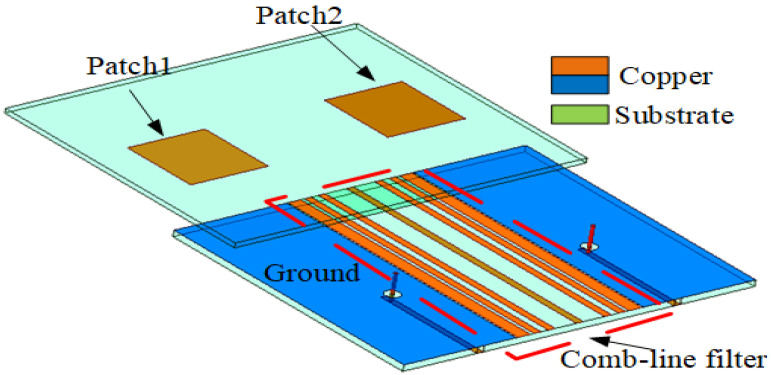
The MIMO antenna with a combline filter.

**Figure 4:**
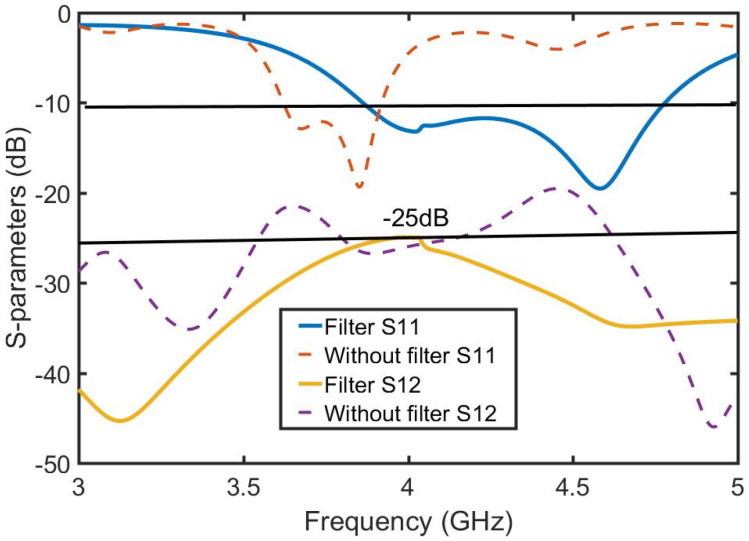
Comparison of S parameters with and without a combline filter.

**Figure 5:**
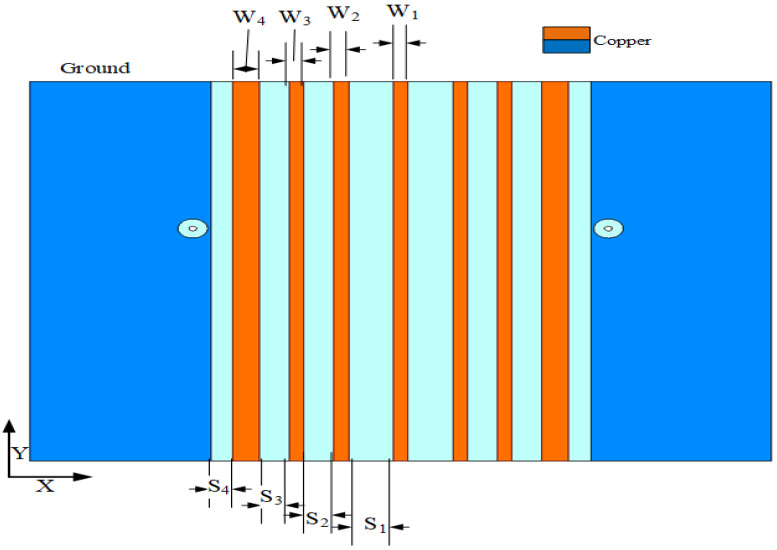
Comber-line filter structure diagram.

**Figure 6:**
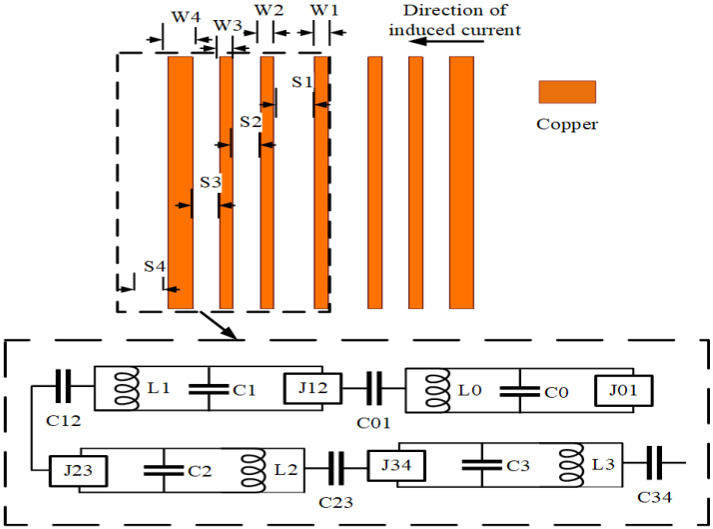
Combline filter equivalent circuit.

As shown in Figure 3, the decoupled MIMO antenna is added to a combline filter at the ground position to form an isolation band. Figure 4 shows the change in the S parameter after adding the combline filter: $S_{11}$ represents the reflection coefficient and $S_{12}$ represents isolation. It is determined that the unit antenna bandwidth of the MIMO antenna increases by 20%, and the coupling decreases by 5 dB. The advantages of this filter are seven microstrip lines and eight microstrip coupling distances, whose structure is simple and does not change the original structure of the antenna, so it minimally affects the antenna radiation and gain. The S parameter is easy to analyze for the symmetrical coupling structure of the combline filter. Figure 5 shows the structure of the combline filter in the ground position of the MIMO antenna, which demonstrates the symmetry of the structure and the parameters to be analyzed. According to the filtering method and principle, we analyze the equivalent circuit diagram formed by microstrip lines in detail in Figure 6.

The microstrip line is composed of an LC circuit and a J converter, and the gap is replaced by $C_{i,i+1}$ for mutual coupling. The specific calculation steps are as follows:

(1) According to Equation (1), the comb filter is a combination of a multiorder filter and a microstrip coupled filter. We set the lower cutoff frequency of the combline filter as $f_{1}$ = 3 GHz ($S_{11}$ < −10 dB), the upper cutoff frequency of the combline filter as $f_{2}$ = 5 GHz ($S_{11}$ < −10 dB), the center frequency of the combline filter as $f_{0}$ = 4 GHz (minimum of $S_{11}$), the roll down coefficient as *a* = −25 dB and the dielectric constant as ϵ = 3.55. The frequency range of the filter and reflection coefficients is from 3 to 5 GHz. We obtain *n* as approximately 7 by calculation.

(2) The electrical length of open-ended microstrip stubs $\theta_{i}$ is calculated through ADS, which is substituted into Equation (3) to obtain the propagation constant $\beta_{i}$ value (*i* = 0, 1, 2, 3).

(3) The microstrip capacitance $C_{i}$ (*i* = 0, 1, 2, 3) and the microstrip coupled capacitance $C_{i,i+1}$ (*i* = 0, 1, 2, 3) in the equivalent circuit are calculated by Equations (4)–(6).

(4) According to Equations (7) and (8), the width of the microstrip $W_{i}$ (*i* = 1, 2, 3, 4) and the coupling distance between microstrip lines $S_{i}$ (*i* = 1, 2, 3, 4) are calculated by the microstrip capacitance $C_{i}$ and the mutual coupling $C_{i,i+1}$. The above calculation roughly describes the combline parameters, and further optimization is executed in Ansys HFSS 2021 software.

(1) $$n=1+\frac{\ln\left(2a/\epsilon \right)-\mathrm{arcosh}\left(\tan\left(\pi f_{1}/2f_{0}\right)/\tan\left(\pi f_{2}/2f_{0}\right)\right)}{\mathrm{arcosh}\left(\sqrt{1+\tan^{2}\left(\pi f_{1}/2f_{0}\right)}/\sqrt{1+\tan^{2}\left(\pi f_{2}/2f_{0}\right)}\right)}$$

(2) $$\mathrm{FBW}=\frac{f_{2}-f_{1}}{f_{0}}$$

(3) $$\beta_{i}{|}_{i=0∼3}=Z_{0}\left(\frac{\cos\theta_{i}+\theta_{i}\csc^{2}\theta_{i}}{2}\right)$$

(4) $$J_{i,i+1}{|}_{i=0∼3}=\sqrt{\frac{\beta_{i}Z_{0}}{g_{i}g_{i+1}}}$$

(5) $$C_{i}{|}_{i=0∼3}=\frac{376.7}{\sqrt{\epsilon }}\left(1-\frac{J_{i,i+1}}{Z_{0}}\right)$$

(6) $$C_{i,i+1}{|}_{i=0∼3}=376.7\sqrt{\epsilon }-C_{i}$$

(7) $$W_{i}{|}_{i=1∼4}=\frac{\mathrm{FBW}}{4\epsilon }\left(C_{i-1}-C_{i-1,i}\right)$$

(8) $$S_{i}{|}_{i=1∼4}=\frac{\mathrm{FBW}}{\pi }\left(\frac{C_{i-1,i}+1}{C_{i-1}+1}\right)$$

where *n* is the filtering order (number of microstrip), $f_{1}$ is the lower cutoff frequency of the combline filter, $f_{2}$ is the upper cutoff frequency of the combline filter, $f_{0}$ is the center frequency of the combline filter, $\theta_{i}$ is the electrical length of the open-ended microstrip stubs, $\beta_{i}$ is the propagation constant, $g_{i}$$g_{i+1}$ is the value of the low-pass filter (LPF) prototype element, $C_{i}$ is the microstrip capacitance, $C_{i,i+1}$ is the microstrip coupled capacitance, $J_{i,i+1}$ is the converter, $W_{i}$ is the width of the microstrip and $S_{i}$ is the coupling distance between microstrip lines [27,28].

**Figure 7:**
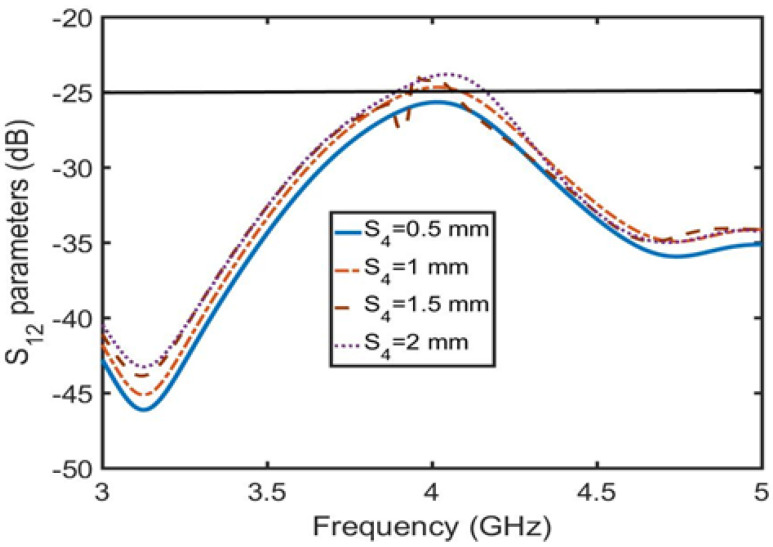
Coupling distance $S_{4}$ slightly adjusts the isolation $S_{12}$.

**Figure 8:**
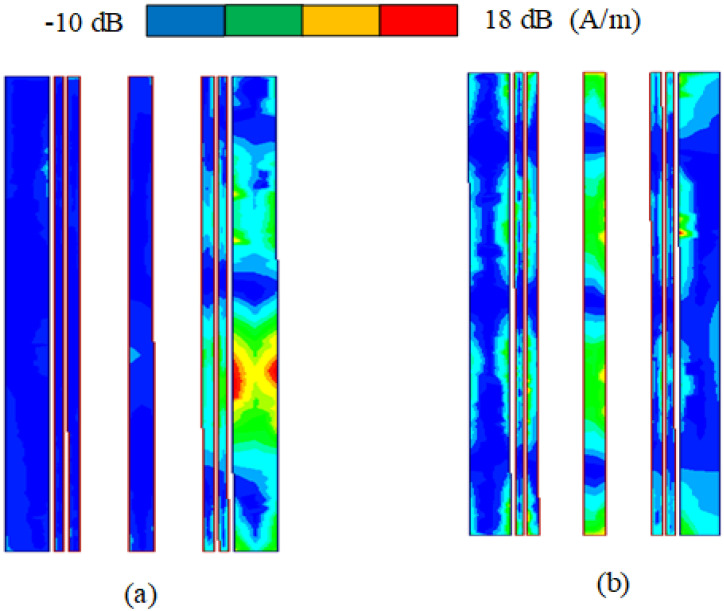
Current intensity distribution of the combline filter at (**a**) 4.5 GHz and (**b**) 4 GHz.

Table 1 shows the results of further optimization by HFSS software. The overall isolation (decoupling) is below −25 dB after optimizing $S_{4}$ in Figure 7. To verify that the combline filter has a decoupling effect, HFSS is used to simulate the current intensity distribution of 4.5 GHz and 4 GHz at the combline filter microstrip line in Figure 8. Figure 8a,b show the current intensity displayed at 4.5 GHz and 4 GHz, respectively. At 4.5 GHz, most of the current is retained in the first half of the filter, and almost no current passes through the second half of the filter. Therefore, when $S_{12}$ = −33 dB, the combline filter has a strong isolation state (decoupling optimal value) and exhibits no blocking current function at 4 GHz when the isolation $S_{12}$ = −27 dB.

## 4. The Absorber Wall Is Used to Reduce the Radiation Coupling of Antenna Elements

**Figure 9:**
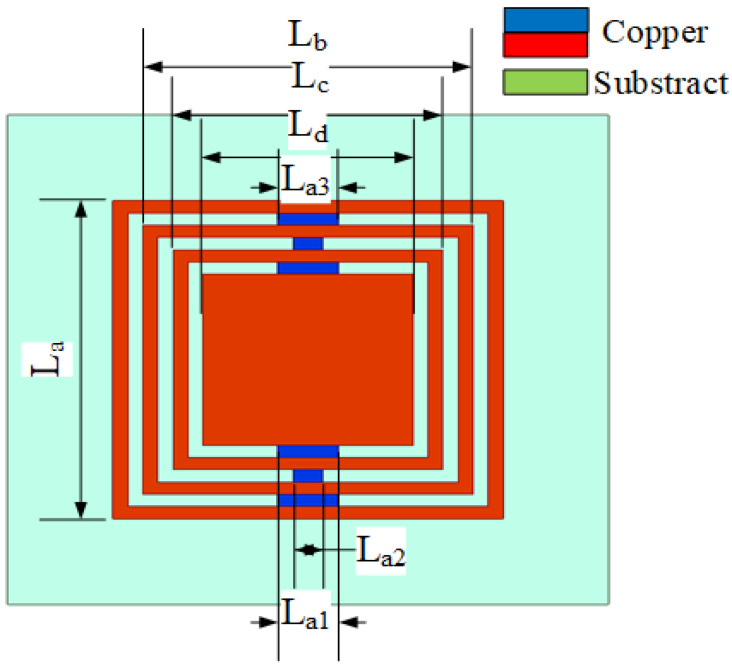
Absorber material unit.

**Figure 10:**
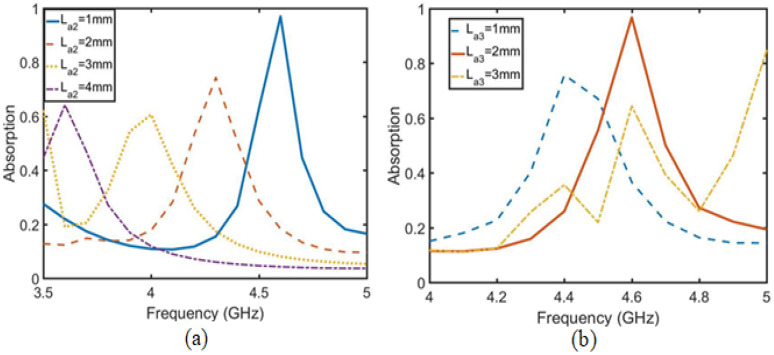
Optimization diagram of unit absorption rate. (**a**) Optimization diagram of parameter $L_{a2}$ and absorptivity. (**b**) Optimization diagram of parameter $L_{a3}$ and absorptivity.

**Figure 11:**
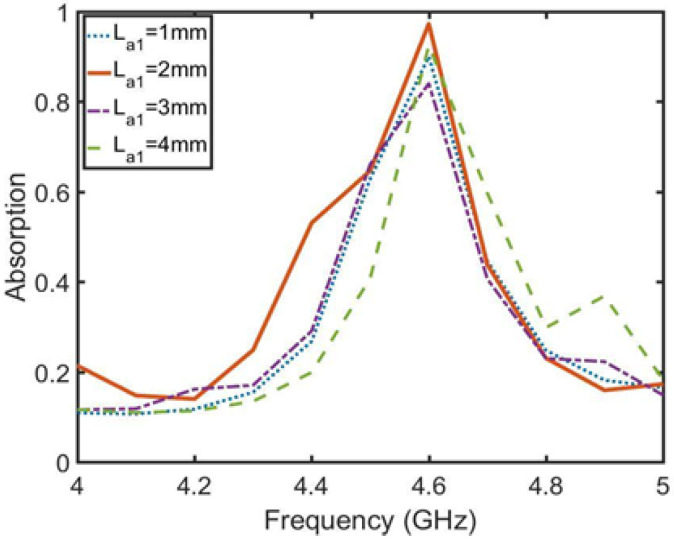
Optimization diagram of parameter $L_{a1}$ and absorptivity.

First, the absorption function of the absorption unit is analyzed, then the relationship between the absorption function of the absorption array and the isolation degree is analyzed and the arrangement distance of the absorption unit is optimized. Finally, the effect of the absorption array is verified by simulation. As shown in Figure 9, the absorption unit is an annular metal embedded with microstrip lines to form an absorption structure. The ring size in the structure is as follows: $L_{a}$ = 13 mm, $L_{b}$ = 11 mm, $L_{c}$ = 9 mm and $L_{d}$ = 7 mm. The embedded microstrip lines $L_{a1}$, $L_{a2}$ and $L_{a3}$ are adjusted to the absorption frequency and absorption rate of the structure. Figure 10a shows that the adjustment of $L_{a2}$ adjusts the absorption frequency, which decreases as the length of $L_{a2}$ increases. Figure 10b shows that $L_{a3}$ is a bidirectional regulation of frequency and absorption. $L_{a1}$ is the length directly affecting the absorption rate in Figure 11. After optimizing the three parameters, when $L_{a1}$ = 2 mm, $L_{a2}$ = 1 mm and $L_{a3}$ = 2 mm, the absorption rate at 4.6 GHz reaches 97%.

**Figure 12:**
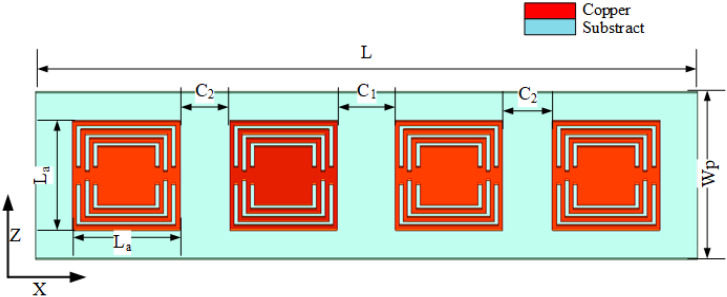
The 1×4 absorption array.

**Figure 13:**
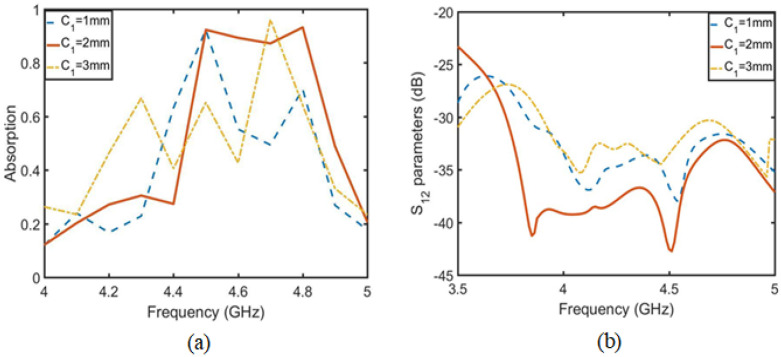
Optimization diagram $C_{1}$ regarding the absorber wall unit distance. (**a**) Relationship between $C_{1}$ and absorption rate. (**b**) Relationship between $C_{1}$ and isolation degree $S_{12}$.

**Figure 14:**
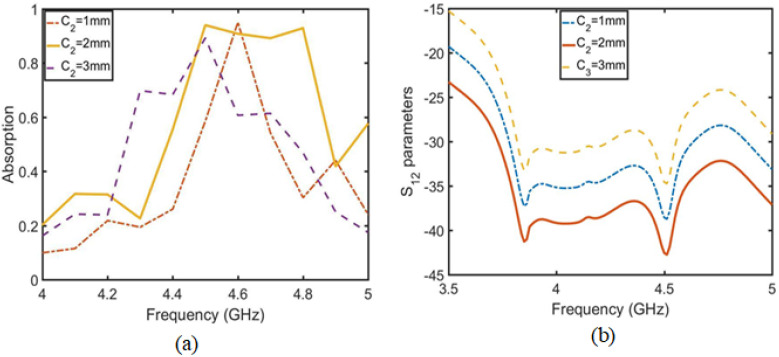
Optimization diagram $C_{2}$ of absorber wall unit distance. (**a**) Relationship between $C_{2}$ and absorption rate. (**b**) Relationship between $C_{2}$ and isolation degree $S_{12}$.

**Figure 15:**
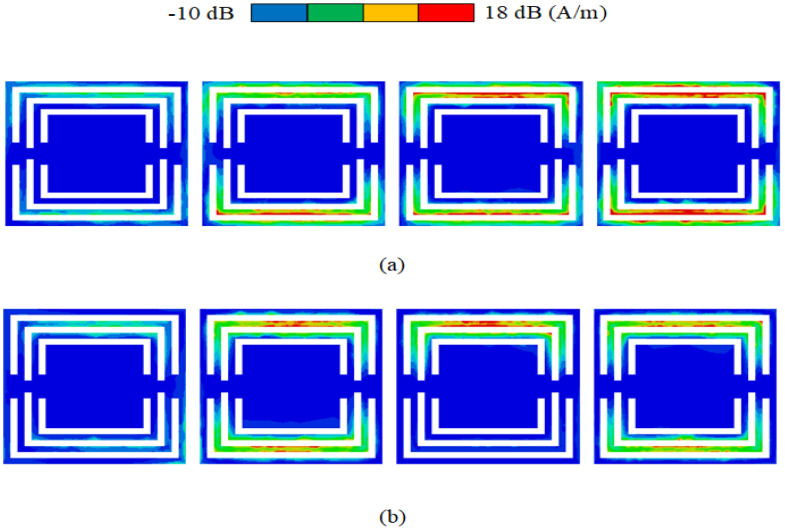
Absorption array current distribution at (**a**) 4.5 GHz and (**b**) 4 GHz.

To further improve the absorption bandwidth, the absorption wall is designed as a 1×4 horizontal array. As shown in Figure 12, the distance between parameters $C_{1}$ and $C_{2}$ is determined to optimize the absorption bandwidth. Figure 13 and Figure 14 show the absorption diagram of the array and the isolation diagram of the MIMO antenna unit, respectively. When $C_{1}$ = 2 mm, the absorption rate is proportional to the isolation degree, which indicates that the greater the absorption bandwidth and absorption rate in Figure 13a, the greater the isolation in Figure 13b. The same principle applies to Figure 14. When $C_{2}$ = 2 mm, the absorption rate is proportional to the isolation degree. The final optimization parameter values are shown by HFSS software in Table 2. The optimized MIMO antenna with an absorber wall and combline filter was simulated, as shown in Figure 15a. When the 4.5 GHz isolation degree $S_{12}$ = −44 dB, the overall absorption current distribution is concentrated in the last three absorption units, while in Figure 15b, the current distribution is concentrated in the two units at 4 GHz, so the isolation degree $S_{12}$ = −39 dB. In this scenario, the absorption rate is proportional to isolation.

## 5. Measured and Simulated Results

**Figure 16:**
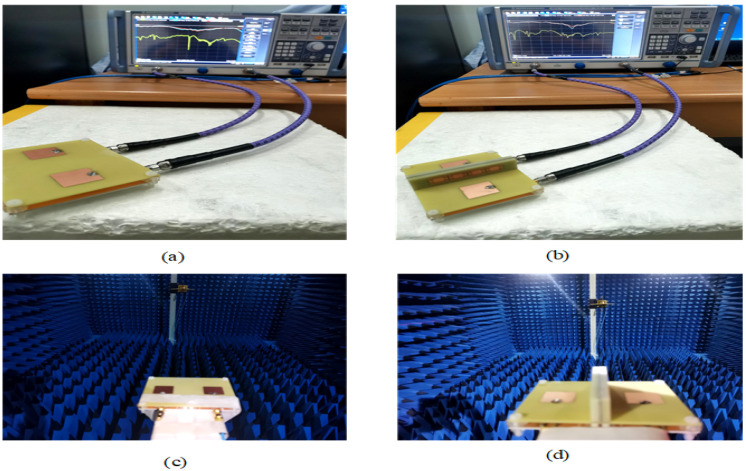
The two MIMO antennas in the S-parameter analysis: (**a**) only the filter structure and (**b**) the filter and absorber structure. The two MIMO antennas in the radiation analysis: (**c**) only the filter structure and (**d**) the filter and absorber structure.

To verify the validity of our design concept, a prototype of the proposed MIMO antenna was fabricated and measured. As shown in Figure 16a,b, an R&S ZNB20 vector network analyzer is used to measure the reflection coefficient and mutual coupling of the two MIMO antennas, for which the insertion loss is less than 0.05 dB. The experimental setup in the anechoic chamber and the two MIMO antennas are shown in Figure 16c,d. The port of the antenna is welded to a 50 ohm SMA. HFSS software was used to simulate the MIMO antenna.

### 5.1. S-Parameter Analysis

**Figure 17:**
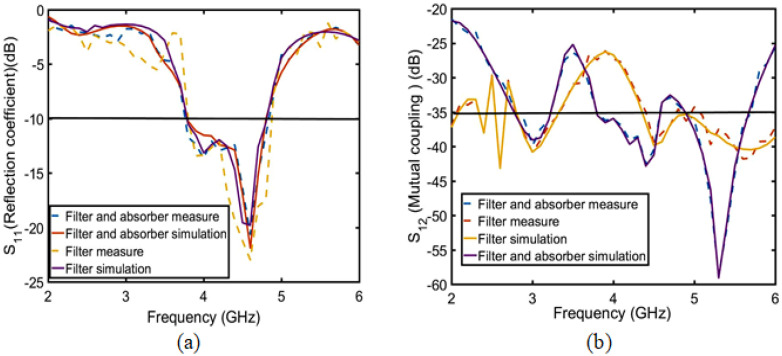
Only the filter structure and the filter and absorption structure S parameters of the simulation and measurement analysis: (**a**) $S_{11}$ and (**b**) $S_{12}$.

Figure 17a shows that the reflection coefficient simulation and measurement results essentially agree, with less than −10 dB in the frequency range of 3.8–4.8 GHz. When the operating frequency is 3.8–4.8 GHz, as shown in Figure 17b, the mutual coupling of the MIMO antennas with the two absorber and combline filter structures is much lower than that of MIMO antennas with only the combline filter structure. In the absorption band of 4.5–4.8 GHz, the mutual coupling is less than −40 dB.

### 5.2. Antenna Radiation Analysis

**Figure 18:**
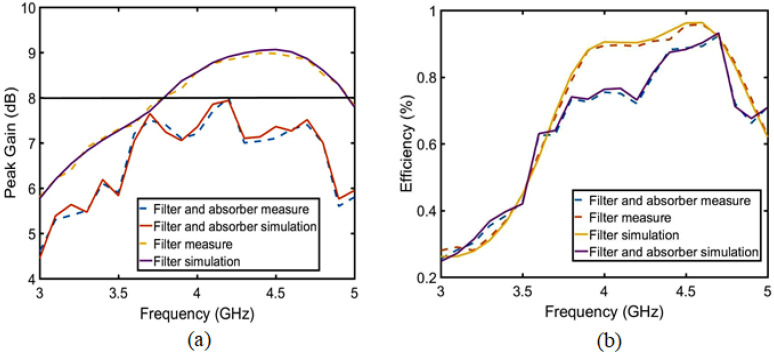
Only the filter structure and the filter and absorption structure radiation of the antenna simulation and measurement analysis: (**a**) peak gain and (**b**) efficiency.

**Figure 19:**
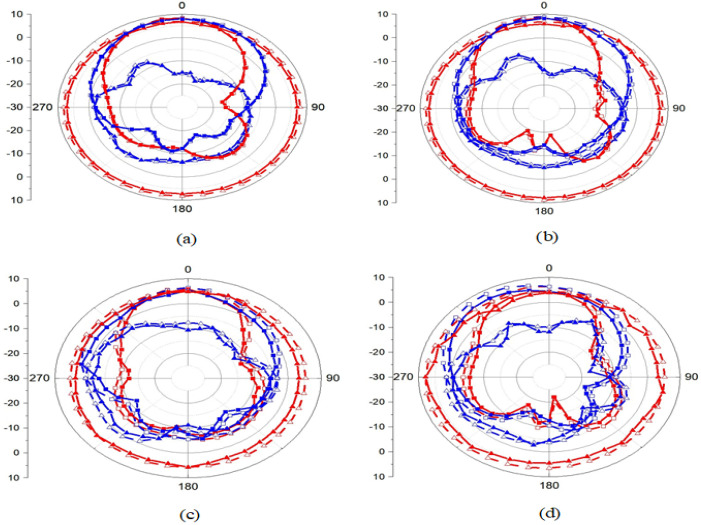
Only the filter structure of the antenna radiation: (**a**) 4 GHz and (**b**) 4.5 GHz. The filter and absorber structure of antenna radiation: (**c**) 4 GHz and (**d**) 4.5 GHz (□ is the co-polarization simulation XOZ, ■ is the co-polarization measure XOZ, □ is the co-polarization simulation YOZ, ■ is the co-polarization measure YOZ, △ is the cross-polarization simulation XOZ, ▲ is the cross-polarization measure XOZ, △ is the cross-polarization simulation YOZ and ▲ is the co-polarization measure YOZ).

The effect of array absorption on the antenna performance is analyzed from three perspectives: gain, radiation efficiency and polarization radiation. As shown in Figure 18a, the antenna gain decreases with the absorption array compared with that without the absorber wall, indicating that the absorber wall absorbs the radiated power of the antenna, resulting in a sharp reduction in the gain. In the absorption band of 4.5–4.8 GHz, the gain is only 7 dB, which is 2 dB lower than that of the MIMO antenna without the absorber wall. Therefore, the radiation efficiency in Figure 18b also decreases. The absorption also has an impact on the antenna radiation polarization. As shown in Figure 19a,b, when the absorber wall is not used for only the combline filter at 4 GHz and 4.5 GHz, both the radiation gain and the radiation direction reach the maximum radiation value at the position of radiation of 0 degrees. After the absorber wall is adopted, the maximum value of the co-polarization radiation is tilted at 15 degrees at 4 GHz, and the cross-polarization angle is almost 90 degrees, as shown in Figure 19c. Similarly, in Figure 19d, the co-polarization is 30 degrees with the plan at 4.5 GHz, and the cross-polarization is also 90 degrees.

### 5.3. Diversity Performance Analysis

**Figure 20:**
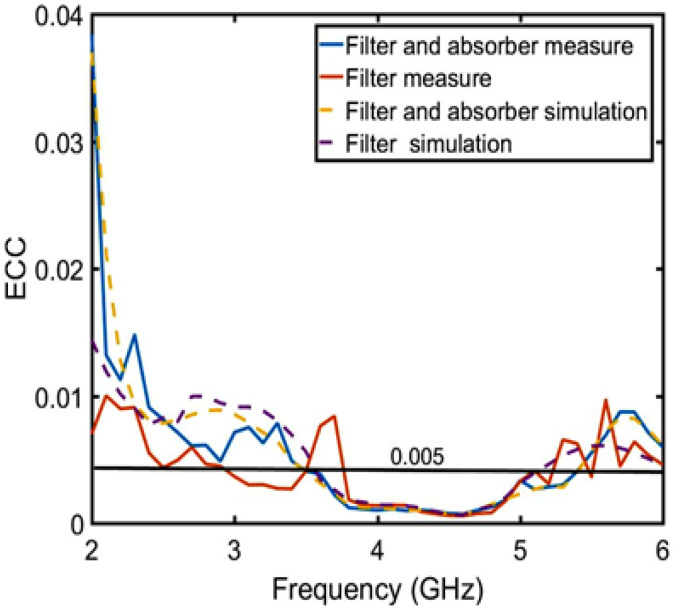
Only the filter structure and the filter and absorption structure ECC of the simulation and measurement analysis.

The envelope correlation coefficient (ECC) is calculated for validating the diversity performance of the antenna, which can be expressed based on the radiation patterns of the antenna in an isotropic propagation environment. The method of ECC calculation is based on the scattering parameters, which can be expressed as

(9) $$\rho_{e}=\frac{|S_{ii}^{*}S_{ij}+S_{ji}^{*}S_{jj}{|}^{2}}{\left(1-|S_{ii}\right){|}^{2}-|S_{ji}{|}^{2})\left(1-|S_{jj}\right){|}^{2}-|S_{ij}{|}^{2})}$$

where $S_{i(j),i(j)}$ (*i*≠*j* and *i*, *j* = 1, 2, 3) represents the complex S-parameters for Port *i* and/or Port *j* and the symbol “*” denotes the Hermitian product [29]. Figure 20 shows the ECC curves calculated by the simulated and measured complex 3D radiation patterns and scattering parameters for adjacent and opposite ports, i.e., ports 1 and 2. It can be seen that the ECC value calculated by the simulated and measured radiation patterns and scattering parameters agree well with each other within the entire band, which are all below 0.005 from 3.8 to 4.8 GHz. In short, the simulated and measured results demonstrate its low correlation for ensuring good channel characteristics.

## 6. Conclusions

In summary, a two-port multiple-input multiple-output (MIMO) antenna from 3.8 to 4.8 GHz is presented for 5G vehicle communication. As shown in Table 3, compared with other MIMO antennas, ours has a moderate overall size, great advantages in bandwidth and gain and improves the isolation degree of adjacent antennas with a smaller ECC. The simulated and measured results show that the proposed MIMO antenna provides an overlapping $S_{11}$ (−10 dB) and a bandwidth of 25% (3.8–4.8 GHz) with a peak gain of 7.8 dBi. Moreover, the fabricated MIMO antenna offers excellent diversity performance, the isolation between antenna elements is very high (>37 dB) and the envelop correlation coefficient (ECC) is lower than 0.005. The demonstrated antenna is a promising candidate for 5G vehicle communication systems for a wide variety of platforms.

## Figures and Tables

**Table 1:** Microstrip line width and coupling distance of the combline filter.

Parameter	Value (mm)	Parameter	Value (mm)
S${}_{1}$	4	W${}_{1}$	2
S${}_{2}$	0.24	W${}_{2}$	1
S${}_{3}$	0.5	W${}_{3}$	0.75
S${}_{4}$	0.5	W${}_{4}$	3.6

**Table 2:** Dimensions of the proposed absorber wall.

Parameter	Value (mm)	Parameter	Value (mm)
L${}_{a}$	13	L${}_{a1}$	2
L${}_{b}$	11	L${}_{a2}$	1
L${}_{c}$	9	L${}_{a3}$	2
L${}_{d}$	7	C${}_{1}$	2
W${}_{p}$	20	C${}_{2}$	2

**Table 3:** Performance comparison with the literature.

Reference	$\lambda_{0}$ × $\lambda_{0}$ × h (mm^2^)	Operating Frequency (GHz)	Maximum Bandwidth (%)	Peak Gain (dBi)	Average Efficiency (%)	Isolation (dB)	ECC	Number of Decoupling Methods
[16]	0.32 × 0.32 × 0.1	2.2–2.5	2.3	3.71	70	>−30	<0.05	1
[17]	1.2 × 1.2 × 0.15	2.4, 3.4	54	6.5	70	>−25	<0.07	1
[18]	0.5 × 0.5 × 1	1.8–2.2	10	3.8	77	>−20	<0.1	1
[19]	1.5 × 1.5 × 0.2	4.7–5.2	10	4	75	>−30	<0.025	1
[20]	2.0 × 2.0 × 0.5	3.6–5.2	20	4	70	>−25	<0.02	1
[21]	2.1 × 2.4 × 0.7	3.8, 5.2	10	4.5	70	>−20	<0.02	1
[22]	2.4 × 2.4 × 0.5	5.2–5.8	12	7	80	>−25	<0.04	2
[23]	0.5 × 0.5 × 0.2	5.5–5.8	6	3.8	72	>−25	<0.03	1
[24]	2.4 × 2.0 × 0.8	3.5, 5.2	6	5.2	68	>−35	<0.05	2
[25]	0.5 × 0.5 × 1.2	2.2–2.8	15	6	78	>−25	<0.05	1
This work	1.1 × 1.2 × 0.3	3.8–4.8	25	7.8	78	>−37	<0.005	2

## Data Availability

Not applicable.
